# Demonstration of Hepatitis C Virus RNA with *In Situ* Hybridization Employing a Locked Nucleic Acid Probe in Humanized Liver of Infected Chimeric Mice and in Needle-Biopsied Human Liver

**DOI:** 10.1155/2013/249535

**Published:** 2013-06-18

**Authors:** Kazuya Shiogama, Ken-ichi Inada, Michinori Kohara, Hidemi Teramoto, Yasuyoshi Mizutani, Takanori Onouchi, Yutaka Tsutsumi

**Affiliations:** ^1^Department of Pathology, Fujita Health University School of Medicine, Toyoake, Aichi 470-1192, Japan; ^2^Department of Microbiology and Cell Biology, Tokyo Metropolitan Institute of Medical Science, Tokyo 156-8506, Japan; ^3^Department of Internal Medicine, Kojin Hospital, Nagoya 463-8530, Japan

## Abstract

*Background*. *In situ* hybridization (ISH) with high sensitivity has been requested to demonstrate hepatitis C virus (HCV) RNA in formalin-fixed, paraffin-embedded (FFPE) sections of the liver. *Methods*. ISH employing a locked-nucleic-acid- (LNA-)modified oligonucleotide probe and biotin-free catalyzed signal amplification system (CSAII) was applied to HCV-RNA detection in the liver tissue. Nested reverse-transcription polymerase chain reaction (RT-PCR) was performed for HCV genotyping using total RNA extracted from FFPE sections. The target tissues included FFPE tissue sections of humanized livers in HCV-infected chimeric mice (HCV genotypes 1a, 1b, and 2a and noninfected) and of needle-biopsied livers from HCV-infected patients. *Results*. HCV-RNA was demonstrated with the ISH technique in HCV-infected liver tissues from both chimeric mice and 9 (82%) of 11 patients with HCV infection. The HCV signals were sensitive to RNase. Nested RT-PCR confirmed the genotype in 8 (73%) of 11 livers (type 1b: 6 lesions and type 2a: 2 lesions). HCV-RNA was not identified in chronic hepatitis B lesions, fatty liver, autoimmune hepatitis, and hepatocellular carcinoma. *Conclusion*. ISH using the LNA-modified oligonucleotide probe and CSAII was applicable to detecting HCV-RNA in routinely prepared FFPE liver specimens.

## 1. Introduction

Hepatitis C virus (HCV) is a single-stranded RNA virus, a member of the Flaviviridae family. Since the first identification of the HCV genome by Choo et al. [1], HCV study has progressed mainly in the field of HCV functional analysis and therapeutic implications. Because of low viral levels in the serum, the diagnosis of HCV infection has been made with a branched chain DNA signal amplification assay and reverse-transcription polymerase chain reaction (RT-PCR) [2–4]. The sensitivity of HCV detection in the serum thus became reproducible and clinically relevant.

Pathologists are often requested to detect viral pathogens within the routinely prepared biopsy tissue samples, and therefore simple and reliable histochemical techniques are needed. Techniques for visualizing HCV localization within diseased hepatocytes have also been developed. Reports have described the detection of HCV in human liver tissue by using immunohistochemistry and *in situ* hybridization (ISH) [4–14], as well as by *in situ* RT-PCR [4, 6, 15–20]. At the moment, HCV detection with immunohistochemistry and ISH is not yet reproducible enough, we believe. *In situ* RT-PCR may demonstrate highly sensitive signals, but with concomitant increase of false positivity. Reliable histochemical techniques for detecting HCV in formalin-fixed, paraffin-embedded (FFPE) liver tissues are needed, particularly for routine diagnostic purpose.

In the present study, we established an ISH technique using a locked nucleic acid (LNA) probe and biotin-free tyramide amplification system (CSAII) for detecting HCV-RNA in FFPE tissue sections of the liver.

## 2. Materials and Methods

### 2.1. Samples

As positive controls, we sampled the humanized liver of HCV-infected chimeric mice [21, 22], and FFPE tissue sections were prepared. The chimeric mice were maintained by M. Kohara, Department of Microbiology and Cell Biology, Tokyo Metropolitan Institute of Medical Science, Tokyo. A total of 13 humanized liver specimens from HCV-infected chimeric mice were used (noninfected 3, genotype 1a-infected 3, genotype 1b-infected 4, and genotype 2a-infected 3).

A total of 11 needle biopsy liver specimens of chronic hepatitis C were culled from the computer file at the Diagnostic Pathology Division of Fujita Health University Hospital, Toyoake, Japan, and FFPE sections were cut. Needle biopsy specimens of hepatitis-B-virus- (HBV-)infected chronic hepatitis, virus-negative fatty liver and autoimmune hepatitis (*n* = 1, resp.), as well as two surgically removed HCC lesions caused by HCV or HBV infection, were also examined. The nonneoplastic part of the liver tissue (chronic hepatitis B) in the HBV-related HCC case was also evaluated. Non-neoplastic liver tissue was scarcely included in the HCV-related HCC case. Hematoxylin and eosin (HE) staining was performed for demonstrating histological features. The activity of inflammation and the degree of fibrosis were evaluated according to the New Inuyama Classification (1996) [23]. Serum HCV-RNA levels in the patients were measured using an RT-PCR assay, the Amplicor HCV Monitor Assay (Roche Diagnostics), and serum HCV subtypes were determined with nested RT-PCR (Roche Diagnostics).

### 2.2. LNA-Modified Oligonucleotide Probe

The sequence of an HCV-common probe was designed according to the previous description [15]. The target was within the HCV 5′-untranslated region (HCV-5′UTR), which is a highly conserved portion in the HCV genome. An LNA-modified oligonucleotide probe labeled with digoxygenin at the 3′-end was prepared in Gene Design Inc., Ibaraki, Osaka, Japan. The nucleotide sequence of the 45-mer probe was 5′-ALtTTLgGGLcGTLgCCLcCCLgCGLaGALcTGLcTALgCCLgAGLtAGLtGTLtGGLgT-3′, in which La, Lt, Lc, and Lg represent LNA monomers corresponding to the bases A, T, C, and G, respectively.

### 2.3. ISH

FFPE sections were dewaxed in xylene and rehydrated in graded ethanol. Endogenous peroxidase activity was quenched with 0.3% hydrogen peroxide in methanol for 60 minutes at room temperature (RT). After rinsing thrice in diethyl-pyrocarbonate- (DEPC-)treated water, sections were digested with 40 *μ*g/mL proteinase K (Roche Diagnostics, Tokyo, Japan) at 37°C for 15 minutes, washed thrice in DEPC-treated water, submerged in 95% ethanol for 1 minute, and air-dried completely. Then, sections were heat-treated for 5 minutes at 95°C on a hot plate and hybridized with 0.01 *μ*mol/L LNA-modified oligonucleotide probe diluted with *in situ* Hybridization Buffer (Enzo Life Science, Bulter Pike, PA, USA) in an incubation chamber overnight at 37°C. After rinsing in 1x saline sodium citrate (SSC) for 30 minutes at 50°C, sections were rinsed twice in 50 mM Tris-HCl-buffered saline (TBS), pH 7.6, at RT. A horseradish-peroxidase-labeled antidigoxygenin antibody (Roche Diagnostics) diluted at 1 : 100 with 1% bovine serum albumin in TBS was applied for 60 minutes at RT. For amplifying signals, fluorescein-isothiocyanate- (FITC-)conjugated tyramide enclosed in CSAII (Dako, Glostrup, Denmark) was applied to the sections for 15 minutes at RT. Finally, a horseradish-peroxidase-labeled anti-FITC antibody equipped in the CSAII kit was reacted for 30 minutes at RT. Reaction products were visualized in 50 mM Tris-HCl buffer, pH 7.6, containing 20 mg/dL diaminobenzidine tetrahydrochloride and 0.006% hydrogen peroxidase. The nuclei were lightly counterstained with Mayer's hematoxylin.

In order to verify the specificity of the ISH technique for HCV-RNA detection, we carried out the treatment with DNase and RNase before the hybridization with the LNA probe on consecutive sections. Namely, either 10 IU/mL DNase I (Wako, Osaka, Japan) or 100 *μ*g/mL RNase (Sigma-Aldrich, St. Louis, MO, USA) was incubated for 30 minutes at 37°C.

### 2.4. Immunostaining Using a Human Specific Cytokeratin 8/18 (CK8/18) Antibody

In order to distinguish mouse hepatocytes from chimeric human hepatocytes in HCV-infected chimeric mice, we utilized a human-specific CK8/18 monoclonal antibody (clone: NCL 5D3, MP Biochemicals, Santa Ana, CA, USA). After blocking endogenous peroxidase activity, hydrated heat-assisted epitope retrieval was employed using a pressure pan cooker (Delicio 6L, T-FAL, Clichy, France) for 10 minutes. Preliminary study revealed that as a soaking solution, 1 mM ethylenediamine tetraacetic acid solution, pH 8.0, was optimal to retrieve the antigenicity. After pressure cooking, sections were left for 30 minutes at RT for cooling. In order for avoiding nonspecific signals in mouse hepatocytes, a Mouse Stain kit (Nichirei Bioscience, Tokyo) was applied before and after the primary antibody incubation. The human-specific CK8/18 monoclonal antibody at a 1 : 100 dilution was incubated overnight at RT. After rinsing in 10 mM phosphate-buffered saline (PBS), pH 7.2, the sections were reacted with a secondary polymer reagent, Novolink (Novocastra, Newcastle, UK). Reaction products were visualized in the diaminobenzidine-hydrogen peroxide solution. Nuclear counterstaining with hematoxylin followed.

### 2.5. HCV Genotyping by Nested RT-PCR

Five slices of 5 *μ*m thickness sections were collected in Eppendorf tubes. Total RNA was extracted from dewaxed sections using RecoverAll Total Nucleic Acid Isolation kit (Applied Biosytems, Austin, TX, USA), according to the manufacturer's protocol, and stored at −80°C until use. Nested RT-PCR for HCV genotyping was performed, as described previously [24].

### 2.6. Ethical Issue

The present study was approved by the institutional ethical review board for clinical and epidemiological investigations at Fujita Health University, Toyoake. The approval number is 12-193. Written informed consent was obtained from each patient.

## 3. Results

### 3.1. HCV-RNA Localization in Humanized Liver of HCV-Infected Chimeric Mice

**Figure 1:**
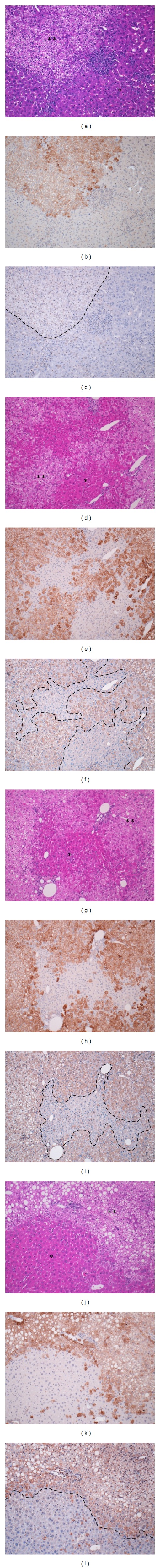
HCV-RNA localization with LNA-based ISH in the liver of chimeric mice infected with HCV of different genotypes. (a)–(c) HCV noninfected, (d)–(f) HCV genotype 1a-infected, (g)–(i) HCV genotype 1b-infected, (j)–(l) HCV genotype 2a-infected, ((a), (d), (g), (j)) HE stain, ((b), (e), (h), (k)) immunostaining for human-specific CK8/18, ((c), (f), (i), (l)) LNA-based ISH, (∗) mouse hepatocytes, and (∗∗) human hepatocytes. HCV-RNA is demonstrated diffusely in the cytoplasm of human hepatocytes in the chimeric livers. No signals are seen in the noninfected liver.

**Figure 2:**
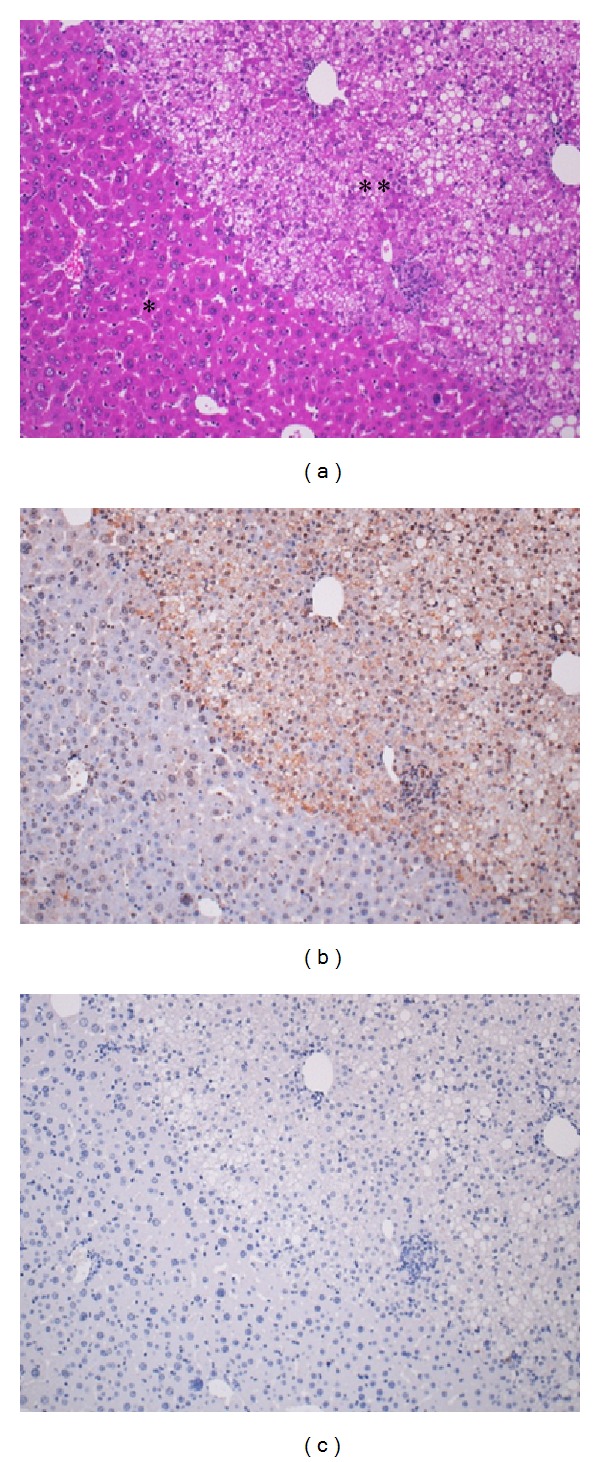
Confirmation of the specificity of LNA-based ISH. (a) HE stain, (b) ISH after DNase treatment, (c) ISH after RNase treatment, (∗) mouse hepatocytes, and (∗∗) human hepatocytes. The mouse hepatocytes are densely eosinophilic, while the human hepatocytes appear pale with deposition of fat droplets. HCV-RNA is observed only in the human hepatocytes in the chimeric liver infected with HCV genotype 1b. The HCV-RNA signals are resistant to DNase treatment but disappear after RNase treatment.

In HE-stained sections, mouse hepatocytes showed densely eosinophilic cytoplasm, while chimeric human hepatocytes were weakly eosinophilic with frequent deposition of fat droplets. Immunoreactivity of human CK8/18 was seen only in human hepatocytes. In all the 10 HCV-infected livers but not the livers of three HCV noninfected mice, ISH demonstrated diffuse cytoplasmic signals in the human hepatocytes. In Figure 1 employing consecutive sections, HCV-RNA was visualized with the ISH technique in human CK8/18-positive chimeric hepatocytes from chimeric mice infected HCV genotypes 1a, 1b, and 2a (in Figure 1, dotted lines encircle the mouse hepatocyte area). No signals were detected in the noninfected chimeric liver tissue. It is of note that HCV-RNA was demonstrated in the cytoplasm of almost all chimeric human hepatocytes. The positive signals remained after DNase treatment but were completely abolished after RNase treatment.  Figure 2 illustrates HCV genotype 1b-infected chimeric mouse liver, showing HE histology and positive cytoplasmic signals, which were resistant to DNase but sensitive to RNase.

### 3.2. HCV-RNA Localization in Needle-Biopsied or Surgically Removed Human Liver Tissues

**Figure 3:**
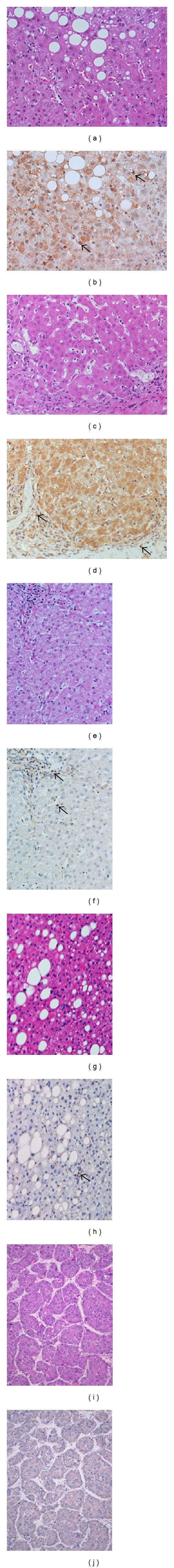
HCV-RNA localization with LNA-based ISH in FFPE biopsy or surgical specimens of the liver. ((a), (b)) Case  10 with chronic hepatitis C, ((c), (d)) case 11 with chronic hepatitis C, ((e), (f)) case 15 with chronic hepatitis B (noncancerous portion of a surgical case for HCC), ((g), (h)) case 13 with fatty liver, ((i), (j)) case 16 with surgically removed hepatitis-C-related HCC, ((a), (c), (e), (g), (i)) HE stain, and ((b), (d), (f), (h), (j)) LNA-based ISH. In HCV-infected human livers, the HCV-RNA signals are demonstrated in a focal/partial pattern (b) or in a diffuse pattern (d). Signals are not demonstrated in the hepatocytes of HBV-infected liver (f), fatty liver (h), and hepatitis-C-related HCC (j). Nonspecific binding of the reagent to Kupffer cells and lymphoid cells is occasionally observed (arrows in the panels (b), (d), (f), and (h)).

Results of the human material are summarized in Table 1. Most of the HCV-positive patients showed high levels of HCV-RNA in the serum. Exceptionally, a surgical case of HCV-related HCC showed a low level of HCV-RNA in the serum. Nested RT-PCR genotyping for HCV using total RNA extracted from FFPE sections was successful in 8 (73%) of 11 biopsied chronic hepatitis C lesions, and in all the 8 cases, the genotypes were comparable with the serum analysis. LNA-based ISH detected signals in 9 (82%) of 11 chronic hepatitis C lesions, including three showing diffuse cytoplasmic positivity, and six showing focal or partial positivity. In three nested RT-PCR negative lesions, ISH revealed negativity (1 lesion) or focal/partial positivity (2 lesions). One lesion was nested RT-PCR-positive and ISH-negative. No HCV-RNA was detected in HBV-related lesions, fatty liver, autoimmune hepatitis, and in HCC lesions.  Figure 3 demonstrates representative histochemical findings. Nonspecific binding of the CSAII reagent to Kupffer cells or lymphoid cells was occasionally observed in both the HCV-infected and HCV-unrelated lesions, as arrows indicate.

## 4. Discussion

We showed herein that LNA-based ISH for HCV-RNA yielded clear cytoplasmic reactivity in infected hepatocytes in FFPE sections. The LNA-modified oligonucleotide probe we employed represents one of the most sensitive probes for ISH [25–29]. LNA-based ISH has been applied to detecting cellular microRNA in FFPE tumor tissues [30–32]. The 45-mer LNA probe was targeted at the HCV-5′UTR containing a sequence common to the HCV subtypes. Positive signals for HCV-RNA were clearly demonstrated in HCV 1a-, 1b-, and 2a-infected humanized livers of chimeric mice. HCV-infected chimeric mice with humanized liver thus functioned as a useful model for histochemically demonstrating the HCV genome, particularly when immunostaining for human-specific CK8/18 was combined.

In needle-biopsied human liver specimens, HCV-RNA was detected in 8 of 11 HCV-infected samples with the nested RT-PCR analysis and in 9 of 11 with the ISH technique. In nested RT-PCR negative cases, ISH was negative or partially positive, suggesting that the detection threshold may depend on the virus load. Diffuse HCV expression pattern in the hepatocytes was already reported by Nuriya et al. [15], Liang et al. [33], and Li et al. [34].

In the present ISH study, HCV-RNA was undetectable in the hepatocytes in HBV-infected livers, nonviral liver disorders (fatty liver and autoimmune hepatitis), and in HCV-related HCC. With ISH using a digoxygenin-labeled cDNA probe, Tang et al. reported positivity in 8 (67%) of 12 HCV-related HCC [12]. It has been documented with an *in situ* RT-PCR technique that HCV-RNA decreases along with the progression from liver cirrhosis to HCC [35]. More cases of HCC need to be analyzed, in relation to the distribution of the HCV genome in cancerous lesions.

Revie and Salahuddin reviewed that HCV replicated in macrophages, B and T lymphocytes, and other nonhepatocellular components [36]. However, we judged the nonhepatocellular bindings as nonspecific reactions, primarily because of the staining in non-HCV cases. Under our present condition, nonspecific binding of the reagents, particularly CSAII, to Kupffer cells and lymphoid cells was observed. Further technical improvement is requested for reliable and reproducible HCV-RNA detection in FFPE specimens.

In conclusion, the ISH technique using the LNA-modified oligonucleotide probe and CSAII can be applied to detecting HCV-RNA in liver biopsy specimens, the sensitivity probably being comparable to *in situ* RT-PCR. For detecting the HCV genome in routinely prepared liver biopsy specimens, our ISH sequence is relatively simple and requires no special equipment to perform. At the moment, this method seems to be suitable for demonstrating HCV genome in FFPE sections and will become a valuable tool for routine histopathological diagnosis of HCV infection using FFPE liver biopsy specimens.

## Figures and Tables

**Table 1:** HCV-RNA demonstration with LNA-based ISH and nested RT-PCR in FFPE human liver tissues.

Patient	Biopsy or surgery	Diagnosis New Inuyama Classification	Serum		FFPE liver tissue		
			HCV-RNA (KIU/mL)	Nested RT-PCR	Nested RT-PCR	ISH	
Case 1	B	Chronic hepatitis C, A2F2	5,1 × 10^2^≦	1b	1b	+	Focal/partial
Case 2	B	Chronic hepatitis C, A1F1	2,8 × 10^2^	1b	1b	+	Focal/partial
Case 3	B	Chronic hepatitis C, A2,F1	2,4 × 10^2^	2a	2a	+	Diffuse
Case 4	B	Chronic hepatitis C, A1F0	1,9 × 10^2^	2a	2a	−	
Case 5	B	Chronic hepatitis C, A1F1	2,5 × 10^2^	1b	1b	+	Focal/partial
Case 6	B	Chronic hepatitis C, A2F3	2,7 × 10^2^	1b	−	−	
Case 7	B	Chronic hepatitis C, A2F1	2,2 × 10^2^	2a	−	+	Focal/partial
Case 8	B	Chronic hepatitis C, A3F3	5,1 × 10^2^	1b	1b	+	Focal/partial
Case 9	B	Chronic hepatitis C, A2F1	2,9 × 10^2^	1b	1b	+	Diffuse
Case 10	B	Chronic hepatitis C, A2F2	3,1 × 10^2^	2a	−	+	Focal/partial
Case 11	B	Chronic hepatitis C, A2F3	3,3 × 10^2^	1b	1b	+	Diffuse

Case 12	B	Chronic hepatitis B, A3F3	Not detected	Not done	−	−	
Case 13	B	Fatty liver, A0F0	Not detected	Not done	−	−	
Case 14	B	Autoimmune hepatitis, A3F3	Not detected	Not done	−	−	
Case 15	S	Hepatitis-B-related HCC Chronic hepatitis B, A2F2	Not detected	Not done	−	− −	
Case 16	S	Hepatitis-C-related HCC	84	Not done	−	−	

New Inuyama Classification (1996).

Activity of inflammation: A1: mild, A2: moderate, and A3: severe.

Fibrosis: F0: none, F1: portal widening, F2: bridging fibrosis, and F3: bridging fibrosis with lobular distortion.
